# Small-World Propensity and Weighted Brain Networks

**DOI:** 10.1038/srep22057

**Published:** 2016-02-25

**Authors:** Sarah Feldt Muldoon, Eric W. Bridgeford, Danielle S. Bassett

**Affiliations:** 1 Department of Bioengineering, University of Pennsylvania, Philadelphia, PA 19104 USA; 2 US Army Research Laboratory, Aberdeen Proving Ground, MD 21005, USA; 3 Department of Mathematics and Computational and Data-Enabled Science and Engineering Program, University at Buffalo, Buffalo, NY 14260 USA; 4 Department of Biomedical Engineering, Johns Hopkins University, Baltimore, MD 21218 USA; 5 Department of Electrical and Systems Engineering, University of Pennsylvania, Philadelphia, PA 19104 USA

## Abstract

Quantitative descriptions of network structure can provide fundamental insights into the function of interconnected complex systems. Small-world structure, diagnosed by high local clustering yet short average path length between any two nodes, promotes information flow in coupled systems, a key function that can differ across conditions or between groups. However, current techniques to quantify small-worldness are density dependent and neglect important features such as the strength of network connections, limiting their application in real-world systems. Here, we address both limitations with a novel metric called the Small-World Propensity (SWP). In its binary instantiation, the SWP provides an unbiased assessment of small-world structure in networks of varying densities. We extend this concept to the case of weighted brain networks by developing (i) a standardized procedure for generating weighted small-world networks, (ii) a weighted extension of the SWP, and (iii) a method for mapping observed brain network data onto the theoretical model. In applying these techniques to compare real-world brain networks, we uncover the surprising fact that the canonical biological small-world network, the *C. elegans* neuronal network, has strikingly low SWP. These metrics, models, and maps form a coherent toolbox for the assessment and comparison of architectural properties in brain networks.

The ability to infer fundamental principles of system function from large sets of interconnected variables is of increasingly pressing importance for structural characterization, functional prediction, and novel network design. Traditionally, network science has been posited as a particularly appealing framework in which to form such inferences based on its simplicity and ubiquitous utility^1^. Yet, it is exactly this simplicity – arguably network science’s greatest strength – which often ignores critical system details, causing it to dual as the field’s greatest potential weakness^2^. For example, many network-based tools were originally developed to examine isolated networks constructed from binary links. However, storage facilities around the world now house rich new data offering multiple instances of the same or related networks, with finely measured weights on links between nodes. Progress in using these data to understand and controllably manipulate complex systems^3^ requires complementary theoretical advances in the realism of network-based analysis tools.

Particularly relevant tools are those used for characterization: quantitative statistics to describe the organization or structure of networks, from which one can infer properties of their function. A quintessential example is the structure of small-worldness, ubiquitous across many real-world networks^4^, whose local clustering combined with the ability to move quickly through the network has been shown to have important implications for functions from synchronizability to information flow^5^,^6^,^7^,^8^. Watts and Strogatz formalized the concept of a small-world network by describing a simple theoretical model on binary networks^9^. Nodes are placed on a lattice and connected to their nearest neighbors within a radius, *r*, giving the network a high clustering coefficient. Shortcuts are then introduced to the network by randomly rewiring each edge with some probability, *p*. For *p* = 0 the network is a lattice, for *p* = 1 the network is random, and for *p* ≪ 1 and *p* > 0, the network is small-world.

While this model has proven useful for theoretical investigations^10^,^11^,^12^,^13^, it does not provide a direct metric to assess small-worldness in observed real-world networks. In fact, the original definition of small-worldness provided by Watts and Strogatz of networks that are “highly clustered, like regular lattices, yet have small characteristic path lengths, like random graphs”^9^, does not provide a quantitative method for assessing small-world structure, even within the theoretical model. If one has observations of a network over a range of size scales, one can determine small-worldness by asking whether the characteristic path length scales as the *log*(*N*) where *N* is the number of nodes in the network^14^. More commonly, however, one has observations of networks at a single size scale, and therefore needs to map real-world data to the theoretical Watts-Strogatz model. Such a mapping can be achieved by a comparison of the observed clustering coefficient and path length to that of random and/or lattice networks^15^,^16^,^17^. Although this mapping of data onto the theoretical model does not take into account other drivers of clustering such as modularity, the practice of mapping real-world data onto the theoretical model is currently held as the preferred methodology for quantifying small-world structure.

Critically, these models – and associated tools and mappings – have lagged behind advances in the data sciences enabling more comprehensive network measurement. Specifically, these statistics are dependent on network density and neglect critical variables such as the strengths of connections between nodes, limiting their ability to diagnose and compare small-world structure in different networks. The lag between the available data and appropriate analytic tools is especially prevalent when examining weighted networks, perhaps due to the intricacies associated with interpreting the physical meaning of weights in different classes of networks. Because weights can have different meanings in different contexts, it is not necessarily true that a single weighted statistic will be appropriate in all contexts, and one must always reflect on the physical interpretation of network weights before choosing the appropriate tool.

The limitations inherent in these methods are critical in the context of many different disciplines, and given the above concerns, we choose to focus our proposed methodology on a single application. Here we examine brain networks, where the comparison of networks with differing densities is particularly salient. For example, neuronal networks imaged at different times in development can have radically different synaptic densities (number of neuron-to-neuron connections), myelination, and other age-related maturation processes^18^,^19^,^20^. Diseases of neurodevelopment, including schizophrenia, are also associated with changes in synaptic density^21^ and alterations in large-scale white matter pathways^22^. Moreover, large-scale brain networks imaged at different stages of atrophy in neurological disorders like Alzheimer’s disease can have drastically different region-to-region connectivity^23^. Additonally, brain networks are a class of weighted networks where edges can represent either structural or functional connections. At the micro-scale, nodes represent individual neurons and structural edges represent the number of connections (synapses or gap junctions) between neurons. At the meso-scale, nodes represent brain regions and structural edges represent the number of streamlines connecting brain regions, which can be estimated from diffusion weighted imaging data. At both scales, one can also study functional networks where edges instead represent statistical relationships quantifying the similarity between nodal dynamics^24^,^25^. We expect that strong and weak connections will differentially contribute to overall network function^26^,^27^, and to ignore this relevant aspect of the network structure could produce misleading results.

To address these limitations, we introduce a novel diagnostic called the Small-World Propensity (SWP), which quantifies the extent to which a network displays small-world characteristics while accounting for variation in network density. We then focus on the application of the SWP to weighted brain networks and present (i) a standardized procedure for generating weighted small-world networks, (ii) a weighted extension of the SWP, and (iii) a stringent and generalizable method for mapping brain network data onto the theoretical model. We verify our methods using standard benchmark networks and use them to examine small-world properties of real-world brain networks in humans and neuronal networks in worms. Surprisingly, we observe that the neuronal network of *C. elegans*, originally touted as a quintessential example of a biological small-world network, shows especially weak SWP.

## Results

### Small-World Propensity

To quantify the extent to which a network displays small-world structure, we define the Small-World Propensity, *ϕ*, to reflect the deviation of a network’s clustering coefficient, *C*_*obs*_, and characteristic path length, *L*_*obs*_, from both lattice (*C*_*latt*_, *L*_*latt*_) and random (*C*_*rand*_, *L*_*rand*_) networks constructed with the same number of nodes and the same degree distribution:


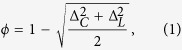


where


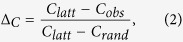


and


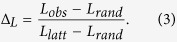


**Figure 1:**
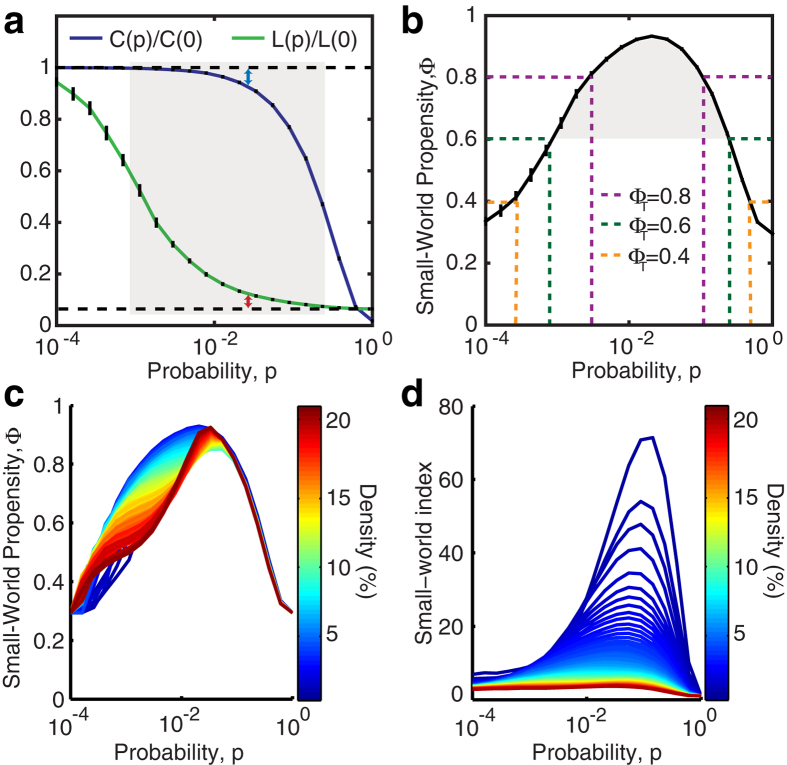
Small-World Propensity in binary networks. (**a**) Clustering coefficient and path length as a function of the rewiring parameter, *p*, for a standard Watts-Strogatz formulation of a small-world network with *N* = 1000 nodes and *r* = 5. The dashed horizontal lines mark the baseline value of the clustering coefficient in a similar lattice network (top) and the baseline value for the path length in a similar random network (bottom). (**b**) SWP calculated for the same network as in panel (**a**). Error bars represent the standard error of the mean calculated over 50 simulations, and the shaded regions represent the range denoted as SW if using a threshold value of *ϕ*_*T*_ = 0.6. (**c**) SWP as a function of network density (increasing *r* for *N* = 1000 nodes). (**d**) Small-world index for the same networks as in panel (**c**).

The ratios Δ_*C*_ and Δ_*L*_ represent the fractional deviation of the metric (*C*_*obs*_ or *L*_*obs*_) from its respective null model (a lattice or random network). See the red and blue arrows in Fig. 1a for a schematic of the deviation, and see the Methods for mathematical definitions of *C* and *L*. Because it is occasionally possible for real-world networks to display path lengths or clustering coefficients that exceed that of a lattice or random network, we bound both Δ_*C*_ and Δ_*L*_ between 0 and 1. Thus, if Δ_*C*_ or Δ_*L*_ > 1, we set Δ_*C*_ or Δ_*L*_ = 1 and if Δ_*C*_ or Δ_*L*_ < 0, we set Δ_*C*_ or Δ_*L*_ = 0, which guarantees that *ϕ* is bounded in the range [0, 1]. Networks with high small-world characteristics (low Δ_*C*_ and Δ_*L*_) will have a value of *ϕ* close to 1, while lower values of *ϕ* represent larger deviations from the respective null models for clustering and path length, and display less small-world structure.

In a standard Watts-Strogatz model, we observe that SWP is maximal for network configurations with the greatest small-world characteristics (Fig. 1a,b). For *p* = 0, we have (Δ_*C*_, Δ_*L*_) = (0, 1) and *ϕ* = 0.29. For small *p*, the SWP remains low, driven by a high path length compared to that of a random network (resulting in a large Δ_*L*_). However, as *p* increases, the path length quickly becomes closer to that of a random network, while the network retains a high clustering coefficient, similar to a lattice. Thus, when the SWP is maximal for *p* ≈ 0.02, we see equal contributions from Δ_*C*_ and Δ_*L*_. As *p* increases further, the network becomes increasingly random: the path length remains small, and the low SWP is now driven by the lack of local clustering (high Δ_*C*_). Finally, for *p* = 1, we have (Δ_*C*_, Δ_*L*_) = (1, 0) and *ϕ* = 0.29 as in the case of *p* = 0.

As illustrated by the application to the Watts-Strogatz model, the SWP is best utilized as a comparative metric to describe the extent to which a network displays small-world (SW) structure. While a definitive hard threshold on this value is meaningful only in the context of a theoretical model^17^, we pragmatically choose a reference value of *ϕ*_*T*_ = 0.6 to distinguish a network with a strong small-world propensity from a network with weak small-world propensity (Fig. 1b). It is also important to stress that although we have suggested *ϕ*_*T*_ = 0.6 as a potentially useful threshold above which a network falls in the range typically identified as SW when applying a Watts-Strogatz characterization, more or less stringent definitions of SW structure could be chosen.

The SWP has the critical ability to discern differences in small-world structure across network densities (Fig. 1c). As density increases, the range of values spanned by the clustering coefficient and path length decreases (Supplementary Fig. 1). The SWP is normalized by these ranges, minimizing the effects of network density on the calculation. The utility of this feature is particularly evident in comparison to the commonly used small-world index, *σ*, proposed by Humphries *et al.*^15^,^16^. As seen in Fig. 1c,d, unlike the small-world index, the SWP retains a large dynamic range even as network density is increased, accurately pinpointing a small-world regime. (See the Supplementary Information for a discussion of small-worldness in high density networks.)

### Contribution to Deviation

**Figure 2:**
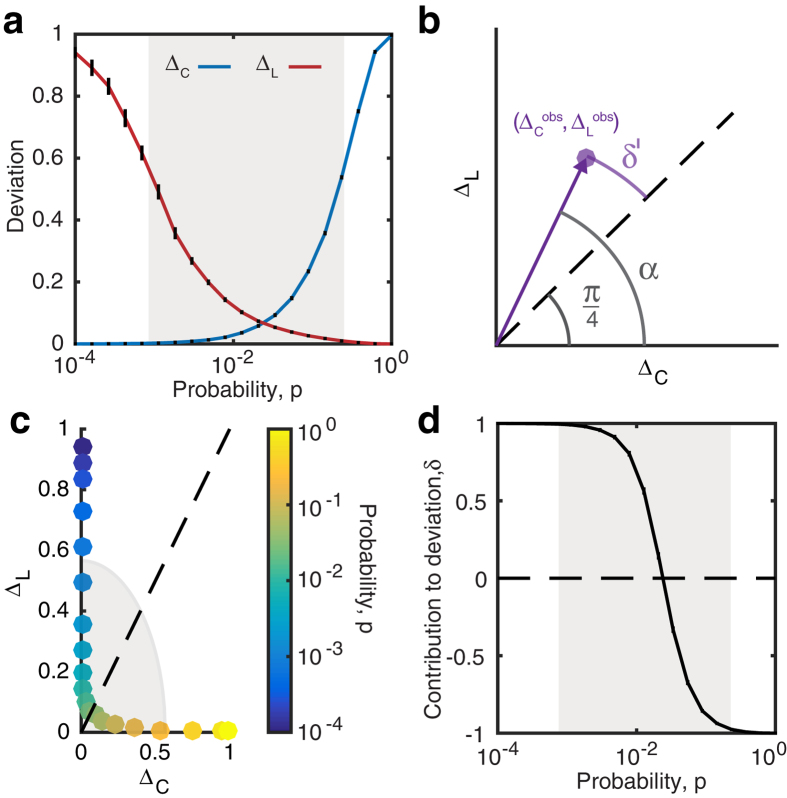
Contribution to Deviation in binary networks. (**a**) Δ_*C*_ and Δ_*L*_ as a function of the rewiring parameter, *p*, for a standard Watts-Strogatz formulation of a small-world network with *N* = 1000 nodes and *r* = 5. (**b**) Schematic depicting a vector describing an observed measurement of Δ_*C*_ and Δ_*L*_, depicted in the (Δ_*C*_, Δ_*L*_) space. The dashed line shows the line of equal contribution between Δ_*C*_ and Δ_*L*_, and *δ*′ depicts the angular deviation of the observed values from equal contribution. (**c**) Observed Δ_*C*_ and Δ_*L*_ values for the standard Watts-Strogatz network depicted in (**a**) shown in the (Δ_*C*_, Δ_*L*_) space. (**d**) The resulting Contribution to Deviation for the Watts-Strogatz transition shown in panels (**a**,**b**). Error bars represent the standard error of the mean calculated over 50 simulations, and the shaded regions represent the range denoted as SW if using a threshold value of *ϕ*_*T*_ = 0.6.

As suggested above, networks with a high SWP can fall into one of three classes: (i) equally high clustering and low path length (both low Δ_*C*_ and Δ_*L*_), (ii) high clustering and moderate path length (low Δ_*C*_ and moderate Δ_*L*_) or (iii) moderate clustering and low path length (moderate Δ_*C*_ and low Δ_*L*_). It is therefore interesting to examine not only the SWP of a network, but also the relative contributions of Δ_*C*_ and Δ_*L*_ to this value. In Fig. 2a, we plot the behavior of Δ_*C*_ and Δ_*L*_ for a standard Watts-Strogatz network. The point of equal contribution from Δ_*C*_ and Δ_*L*_ (which occurs at the intersection of the lines) corresponds to the peak value of SWP in Fig. 1b. To the left of this value, the deviation from path length, Δ_*L*_, reduces the overall SWP value, while to the right of this value, the reduction in SWP is driven by a higher Δ_*C*_. We quantify the extent to which Δ_*C*_ or Δ_*L*_ drive the SWP value by calculating the angular difference of the observed values from the line of equal contribution in the (Δ_*C*_, Δ_*L*_) plane (*δ*′ in Fig. 2b). Mapping this value to the interval [−1, 1], we define the the *contribution to deviation*:


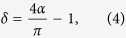


where 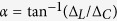 is the angle of the observed (Δ_*C*_, Δ_*L*_) vector. Thus, when the deviation of the observed path length from its null model is maximal (Δ_*L*_ = 1), we find *δ* = 1, whereas when the deviation of the clustering coefficient from its null model is maximal (Δ_*C*_ = 1), we find *δ* = −1. For the standard Watts-Strogatz model, we therefore observe a smooth transition from *δ* = 1 to *δ* = −1 as the network is rewired, with the point of highest SWP occurring when *δ* = 0 (Fig. 2c,d).

### Generating Weighted Small-World Networks

In many real-world systems, interactions between network nodes are best described by weighted connections where the weight of the connection indicates the strength of interaction between network nodes or vertices. It is therefore desirable to extend the SWP to the weighted regime to quantify small-world structure in these systems. To do so, it is necessary to define models of weighted lattice networks and weighted random networks. However, the choice of how one defines these weighted null models is not trivial, and must be based on knowledge of the observed system of interest. Here, we focus our efforts on developing weighted null models for brain networks^28^,^29^, where network nodes represent physical points in space (neurons or brain regions) and connections indicate either the number of structural connections (structural brain networks) or the strength of statistical relationships between the activity time series of network nodes (functional brain networks).

One important feature of brain networks is the fact that these networks are spatially embedded^30^, and the weight of an edge is inversely correlated with the physical distance between nodes: nodes located near one another tend to be linked by stronger edges than nodes located far from one another^28^,^31^,^32^,^33^,^34^. Based on this evidence supporting a strong relationship between physical space and edge strength, we define a weighted lattice network along with a re-wiring mechanism that allows the network to be manipulated from a lattice network to a random network while maintaining the distribution of edge strengths:

1. Begin with a network of *N* nodes that are arranged on a lattice and connected to all neighbors within a radius, *r*.

2. Assign edge weights *w*_*ij*_ according to the distance between nodes *d*_*ij*_:


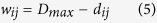


where *D*_*max*_ = *max*{*d*_*ij*_} + *ε* is the maximum possible distance between two nodes plus a single unit of measurement of the lattice spacing. For a lattice reflecting real data, *ε* would equal the precision of the measurement. The inclusion of *ε* ensures that no edges will be assigned a weight of 0.

3. Re-wire each edge with probability, *p*, retaining the weight of the edge.

**Figure 3:**
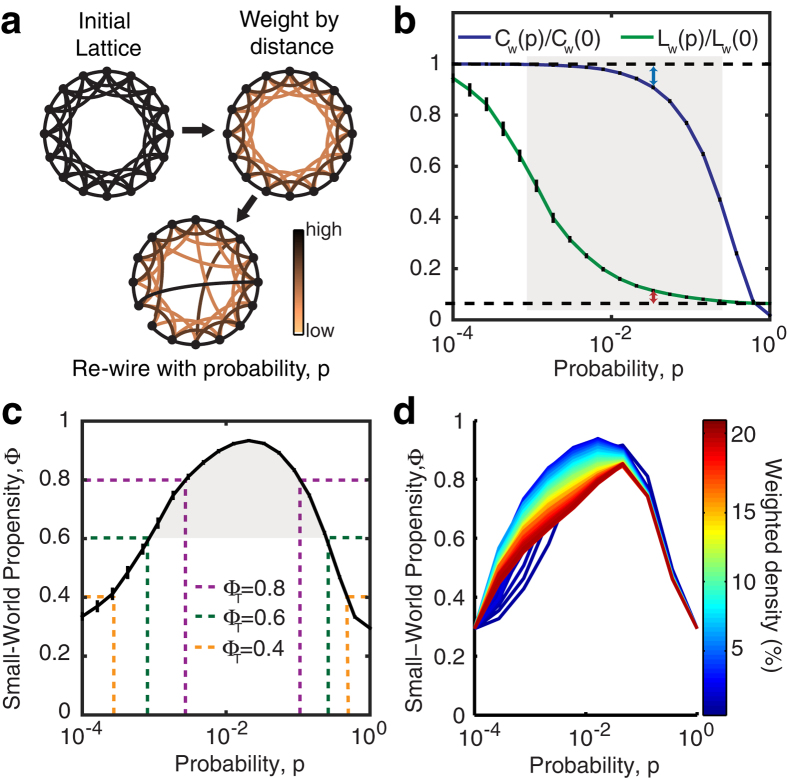
Small-World Propensity in weighted networks. (**a**) Generation of weighted small-world networks. After building a lattice, the edges are weighted by distance such that close edges have a higher strength than distant edges. These edge weights are then retained as links are rewired with a probability, *p*, to create a weighted small-world network. (**b**) Weighted clustering coefficient and weighted path length as a function of the rewiring parameter, *p*, for a weighted formulation of a small-world network with *N* = 1000 nodes and *r* = 5. (**c**) Weighted SWP calculated for the same network as in panel (**b**). Error bars represent the standard error of the mean calculated over 50 simulations, and the shaded regions represent the range denoted as small-world if using a threshold value of *ϕ*_*T*_ = 0.6. (**d**) Weighted SWP as a function of network density (increasing *r* for *N* = 1000 nodes).

**Figure 4:**
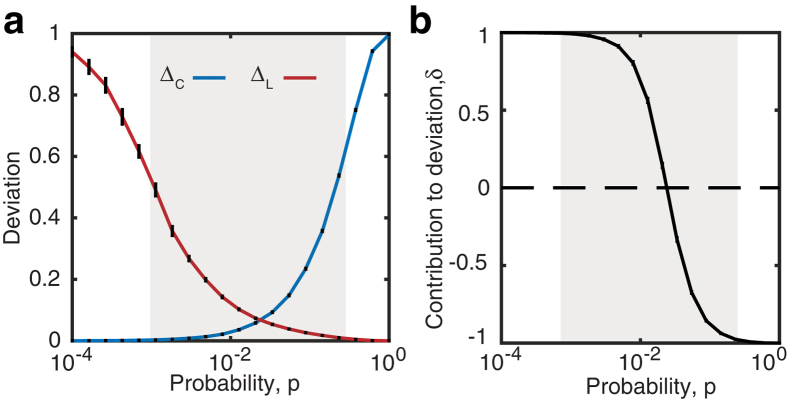
Contribution to Deviation in weighted networks. (**a**) Δ_*C*_ and Δ_*L*_ computed from weighted metrics as a function of the rewiring parameter, *p*, for a standard Watts-Strogatz formulation of a small-world network with *N* = 1000 nodes and *r* = 5. (**b**) The resulting Contribution to Deviation for the weighted Watts-Strogatz transition shown in panel (**a**). Error bars represent the standard error of the mean calculated over 50 simulations, and the shaded regions represent the range denoted as SW if using a threshold value of *ϕ*_*T*_ = 0.6.

Following network formation, we assess small-world structure as a function of the rewiring probability by computing weighted versions of the clustering coefficient, *C*_*w*_, and characteristic path length, *L*_*w*_ (see Fig. 3b and the Methods for definitions). As discussed in depth in the Methods, because here we are interested in information flow on our network, the calculation of *L*_*w*_ is based on the cost associated with moving between network nodes, which we define to be inversely proportional to the edge weight. The clustering coefficient and characteristic path length monitor a transition of our weighted networks through the small-world regime in a manner that is similar to that observed in the original Watts-Strogatz procedure for binary networks (compare Figs 1a and 3b). We quantify this transition by computing a weighted version of the SWP using *C*_*latt*_ = *C*_*w*_(*p* = 0) and *L*_*rand*_ = *L*_*w*_(*p* = 1). We observe that the weighted SWP behaves similarly to the unweighted SWP (compare Figs 1b and 3c), and maintains a large dynamic range as network density is increased (Fig. 3d). Additionally, the behavior of Δ_*C*_, Δ_*L*_, and *δ* track the behavior displayed in the binary case (compare Figs 2b,c and 4).

### Mapping Real-World Observations to Theory

To compute the SWP in a real-world brain network, it is necessary to determine the appropriate comparable lattice and random networks. To account for the effects of network density, we construct lattice and random networks that maintain the network density – number of nodes and edge weight distribution – observed in the real-world network. Specifically, we construct a comparable weighted lattice by arranging the observed edge weights such that the edges that correspond to the smallest Euclidean distance between nodes are assigned the highest weights (see Supplementary Fig. 2 and the corresponding discussion). For example, for a 1D lattice with *N* nodes and unit spacing between nodes, we have *N* edges with a Euclidean distance of *d* = 1 between nodes. We therefore rank the observed edge weights by decreasing strength and randomly distribute the connections with the *N* highest weights among the edges representing *d* = 1. We proceed to distribute the next *N* − 1 edges of highest weight among the edges of the lattice corresponding to *d* = 2, and we continue to proceed in this manner until the total number of edges in the real-world network have been placed in the lattice. To create a comparable random network, the observed edge weights are randomly distributed among the *N* nodes of the network. These reference networks are then used to calculate the SWP of the observed network.

### Synthetic Benchmark Networks

**Figure 5:**
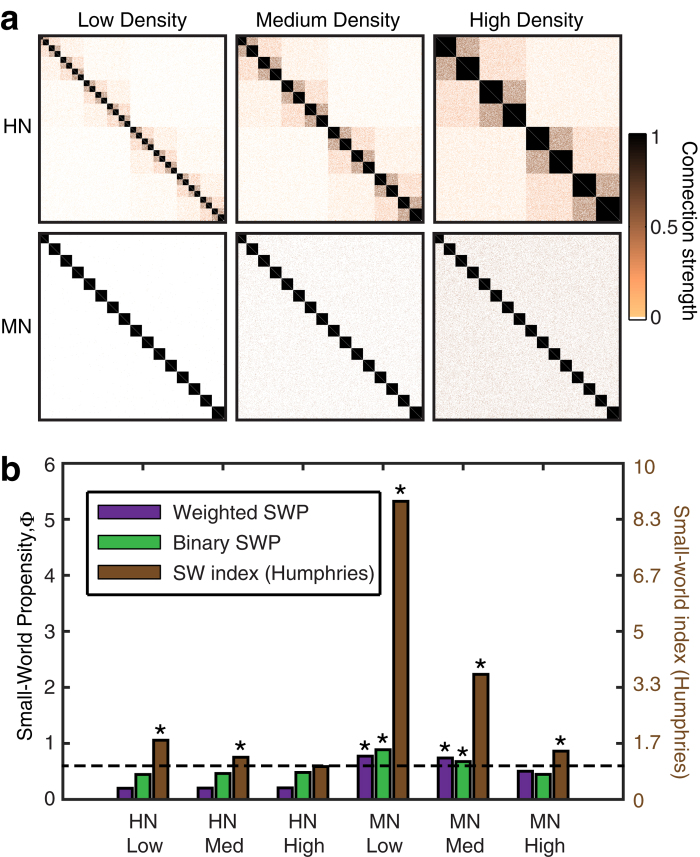
Benchmark networks. (**a**) Adjacency matrices depicting network organization for increasingly dense Hierarchical Modular Networks (HN) and Modular Networks (MN). (**b**) Comparison of the unweighted SWP, weighted SWP, and small-world index when applied to the matrices in panel (**a**). The dashed line represents the chosen threshold for indicating small-world structure (*ϕ*_*T*_ = 0.6 for SWP or *σ*_*T*_ = 1 for the small-world index). Stars denote networks classified as small-world.

To further assess the validity of the SWP, we examine its performance on weighted networks with well studied structure: hierarchical modular networks (HN) and modular networks (MN) (see Methods). While both types of networks have a modular structure and therefore dense local clustering, the networks differ in terms of their path length: the MN incorporate random shortcuts of moderate connection strength between modules, decreasing the weighted path length, while the HN are composed of hierarchically organized modules that are weakly interconnected, increasing the weighted path length (see Fig. 5a). It follows that MN have greater small-world structure than HN.

In Fig. 5, we compare the ability of three metrics to detect small-world structure in these networks constructed at various densities: (i) the weighted SWP computed on the true networks, and (ii) the un-weighted SWP, and (iii) Humphries’ small-world index computed on binarized versions of the true networks. In the HN, we observe that both the weighted and unweighted SWP values are relatively independent of density, while the small-world index is strongly dependent on density. Moreover, the small-world index classifies low and medium density HN as having SW properties (*σ* > 1), despite the long path lengths characteristic of these networks. The SWP instead reflects our expectations and remains below the small-world threshold (*ϕ*_*T*_ = 0.6), even in the case of binarized networks. Importantly, when edge weights are retained, the SWP reveals that the HN are quite different than their MN counterparts and do not show a small-world structure.

In the MN, we observe that the SWP values are sensitive to a meaningful change in the network structure as density increases. Specifically, the path length becomes closer to that of a random network, but the addition of shortcuts between modules reduces the clustering coefficient thereby increasing the deviation from the clustering coefficient of a comparable lattice network Δ_*C*_. This change is reflected by decreasing SWP. The small-world index is less sensitive to the deviation in clustering and classifies all MN as having small-world structure.

### Real-World Brain and Neuronal Networks

**Figure 6 f6:**
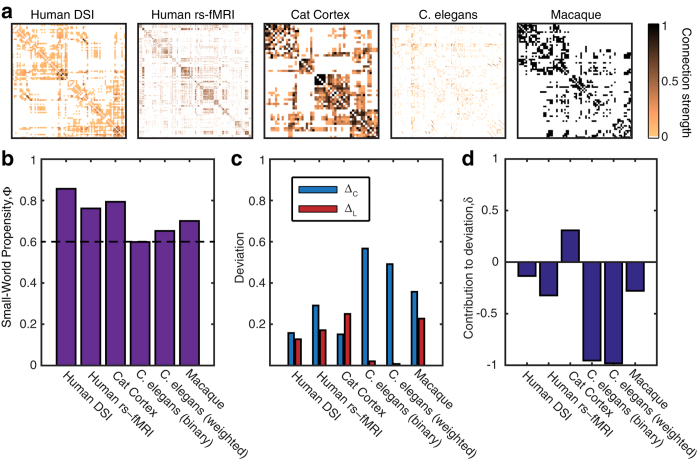
Real-world brain networks. (**a**) Adjacency matrices for four weighted brain networks and one binary brain networks. All matrices are symmetric and values are scaled to be within [0, 1]. The *C. elegans* network is analyzed as both a binary and weighted matrix. (**b**) SWP for the matrices shown in panel (**a**). The dashed line denotes the *ϕ*_*T*_ = 0.6 threshold. (**c**) Breakdown of the SWP into the individual contributions from the clustering coefficient and path length. High values of Δ_*C*_ or Δ_*L*_ indicate a large deviation from the comparable benchmark value, which results in a reduction of the SWP. (**d**) The Contribution to Deviation, *δ*, which summarizes the role of Δ_*C*_ and Δ_*L*_ in driving the SWP calculation. The high values of Δ_*C*_ for both the binary and weighted analysis of the *C. elegans* network are reflected the corresponding large negative values of *δ*.

The quintessential example of a small-world network in biology is the neuronal network of *C. elegans*^9^. Indeed, brain networks more broadly have been described as small-world for the past decade^35^,^36^. Yet, with advanced imaging and methodological techniques that provide more detailed measurements of these data with varying network densities and fine-scale estimates of edge strength, the assumptions of small-worldness have been brought into question^37^. Here we aim to resolve this controversy by applying the SWP to a representative set of structural and functional brain networks from several species whose properties have been well-studied in previous literature, and whose edges are both binary and weighted (Fig. 6a). The four weighted networks are (i) a structural network representing the density of streamline counts connecting 83 brain regions obtained from human diffusion spectrum imaging (DSI) data^3^, (ii) a functional network given by correlations between the blood oxygen level dependent signal of 638 brain regions measured using resting state functional magnetic resonance imaging (rs-fMRI)^38^, (iii) a structural network of the cat cortex obtained from tract-tracing studies between 52 brain regions^39^, and (iv) the neuronal network of *C. elegans* representing the total number of synaptic and gap junction connections between 279 neurons^40^. We additionally study the *C. elegans* neuronal network in its binary representation for comparison with prior studies that have used the binary representation of this network, as well as a binary structural network derived from tract-tracing studies between 71 regions in the macaque cortex^41^.

We observed that all networks displayed relatively large SWP values, indicating that brain and neuronal networks in general do indeed display small-world properties (Fig. 6b). Surprisingly, the network with the lowest SWP, sitting just below the *ϕ*_*T*_ = 0.6 threshold, is the binary representation of the neuronal network of *C. elegans*, the quintessential example of a small-world network in biology^9^. While the SWP for this network rises slightly when the number of connections is included, we find that the *C. elegans* network continues to display the lowest SWP of all brain networks studied. We explored this surprising result by examining the contributions of the clustering (Δ_*C*_) and the path length (Δ_*L*_) to the SWP value (Fig. 6c). In contrast to the other brain networks studied, the neuronal network of *C. elegans* displays drastically different contributions: a remarkably high Δ_*C*_ indicating divergence from a lattice network, and an exceptionally low Δ_*L*_ indicating similarity to a random network. The observed SWP (for both binary and weighted networks) is therefore driven almost entirely by the short path length, indicating that, in reality, this canonical example of a small-world network does not strongly embody small-world principles.

## Discussion

Complex interconnected systems are being increasingly queried for fundamental principles of structure and dynamics, and the ability to characterize, manipulate, and compare these systems requires the development of network-based analysis tools that accurately account for the features of the data. Here we offer a novel network statistic (SWP) that accurately quantifies small-world structure in networked systems, that is agnostic to nuisance variables such as density, and is exquisitely sensitive to critical variables such as the strengths of connections between nodes. Additionally, we focused on neural systems as particularly critical examples where this statistic could be expanded to the weighted regime in order to study real-world data. This enterprise has required the development of (i) a standardized procedure for generating weighted small-world networks, (ii) a weighted extension of the SWP, and (iii) a stringent and generalizable method for mapping real-world data onto the theoretical model. We have illustrated the application and utility of these methods in the context of both benchmark and weighted data from real-world brain networks. The work represents a single effort in the much larger space of meeting the demands of real-world data with increasingly sophisticated network-based tools.

### Small-World Statistics for Real-World Data

The small-world index^15^,^16^ is the most common statistic to quantify small-world structure in binary networks, but produces values greater than one for a large range of network topologies, obscuring accurate interpretations. Another measure of small-worldness has therefore been proposed by Telesford *et al.*^17^ that instead normalizes the clustering coefficient by that of a lattice (as opposed to a random) network, and has been applied to neuroimaging to examine small-world structure^42^,^43^,^44^. By comparing the clustering coefficient to that of a lattice and restricting the values of the measurement to be within [−1, 1], the measure by Telesford *et al.* is able to more accurately discern the small-world regime^43^, but remains density dependent and cannot be applied to weighted networks, making comparison between real-world weighted data sets difficult^42^. Further recent work has used weighted clustering coefficients and path lengths to compare observed networks to random networks^45^, and to measure local small-world structure^46^ and topological dimension. However, the generalizability of these methods has been hampered by the lack of a theoretical model to construct weighted small-world networks and to study the transition in and out of the small-world regime. (Although, see Zhang *et al.*^47^ for a method to generate weighted scale-free small-world networks.) Our work directly fills this gap in the field of neuroscience, allowing the creation of weighted small-world networks with varied degrees of SWP that can be used to compare and contrast network behavior and dynamics throughout the small-world regime.

### Model Assumptions for Weighted Networks

To extend the SWP to weighted brain networks, it was necessary to develop a theoretical model of a weighted lattice. The choice of an appropriate null model is a common problem in network analysis in general^48^, because when mapping real-world data onto a theoretical model, one must always question if the choice of null model reflects the properties of the data. The model that we have chosen to employ here is based on the observation that in brain networks, nodes that are closer in physical space also tend to have a higher connection strength^28^,^31^,^32^,^33^,^34^. The importance of incorporating this information into null models for neuroimaging data is self evident as the choice of null model will clearly influence the interpretation of network statistics (see for example^32^,^49^,^50^ for a discussion of choosing the appropriate spatial null model to calculate motif expression in *C. elegans*). While we have focused our efforts on different types of neural data, the observation of an inverse correlation between physical distance and edge strength has also been made in other physical systems such as human mobility networks^51^ and force chain networks in granular media^52^, indicating that this choice of a weighted null model could also be applicable when studying systems outside of the field of neuroscience. However, great care should be taken to insure that the system of interest meets the modeling assumptions.

### Binary Categories vs. Continuous Narration

Recent network-focused efforts in the applied mathematics, physics, computer science, and engineering communities evidence the age-old tension to retain model simplicity while maximizing pragmatic utility. Network statistics are no exception: we often wish to obtain binary categorizations rather than continuous descriptions. For example, one might wish to emphatically state that a network does or does not display community structure, rich-club architecture, or small-worldness. Yet, arguably a more interesting and useful statement might assess gradations of these properties in real-world systems^48^,^53^. The true power of the SWP lies not in its ability to define small-world structure, but instead in its ability to quantify and compare the *continuous degree* of small-world structure between different networks. Our work therefore complements ongoing efforts to extend traditionally categorical distinctions to continuous measurements in the context of core-periphery structure^53^ and community structure^54^.

### Pragmatic Utility in Neuronal and Brain Networks

Network statistics that are independent of density are critical for network comparisons in real-world settings. In the context of brain networks, the need for such statistics is underscored by the growing interest in examining neuronal networks across (i) time in development and normal aging^18^,^19^, (ii) health and disease^21^,^22^, and (iii) different stages of neurodegeneration^23^. In these and similar contexts, it becomes very difficult to determine if observed differences in small-world structure are simply a function of the differences in network density, or if they represent a true form of topological reorganization. By minimizing the effects of network density in the computation of the SWP, we allow for a more direct comparison of the topological network structure across time, and between groups.

Network statistics that are highly sensitive to edge weights provide increased sensitivity to network function. In the context of brain networks, the need for such statistics has only recently been actively appreciated, as a growing body of literature demonstrates that both healthy and diseased brain function is differentially driven by strong *versus* weak connections^26^,^27^. Weak connections have traditionally been ignored because of commonly applied thresholding techniques^26^, but have recently been identified as potential biomarkers in psychiatric pathologies^26^ and as predictors of cognitive function and fluid intelligence^55^,^56^,^57^. In the future, it will be interesting to build on weighted network diagnostics like the SWP to provide novel quantifications of weak connectivity and its role in cognition.

### Surprising Biological Insights

The canonical example of a biological small-world network is the wiring diagram of *C. elegans*. However, the observation of small-world structure in this organism has been built on a simplification of the weighted wiring diagram to a binary graph. For this reason, we studied both binary and weighted versions of this canonical network. However, even when using the weighted SWP, we observe that in fact, this network displays very little small-world propensity, predominantly due to a lack of local clustering. We speculate that several key biological features of this network may explain this surprising contradiction to the historical literature. The wiring diagram of *C. elegans* is (i) a micro-scale network (nodes represent neurons as opposed to meso-scale brain regions), (ii) represents neuron-to-neuron connections throughout the entire body of the organism rather than only the head, and (iii) is drawn from a comparatively simplistic organism, evolutionarily speaking^33^. These features of scale, physical extent, and evolutionary class may drive topological properties away from the small-world architecture to enable a different class of neural functions than those associated with the brains of higher-order animals. Future work must more stringently examine other canonical examples of small-world networks in diverse real-world systems, and thereby build a more accurate assessment of the role of topology in complex system function.

## Methods

All analysis was done using MATLAB (MathWorks) and code to compute the SWP in real-world networks can be accessed at http://www.seas.upenn.edu/~dsb/.

### Clustering coefficient

To calculate the clustering coefficient, as in^9^, we first calculate the local clustering coefficient for each node, *c*_*i*_ and then define the clustering coefficient, *C*, to be the average of the local coefficients


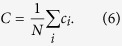


For binary networks, the local coefficient, *c*_*i*_, of each node, *n*_*i*_, is the fraction of closest neighbors of *n*_*i*_ that are also connected. Multiple extensions of *c*_*i*_ to weighted networks have been proposed:

Onnela *et al.*^58^:


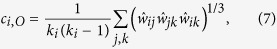


Barrat *et al.*^59^:


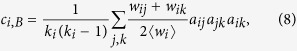


and Zhang *et al.*^60^:


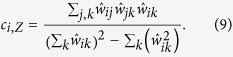


In these equations, *w*_*ij*_ is the strength of a connection between nodes *i* and *j*, 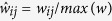, *k*_*i*_ is the number of edges connected to *n*_*i*_, and *a*_*ij*_ = 1 if a connection exists between nodes *i* and *j* (*a*_*ij*_ = 0 otherwise).

Each method of computing the weighted clustering coefficient has been designed to detect a specific feature of weighted clustering^61^,^62^. The measure by Onnela *et al.* is based on subgraph intensity, the measure by Barrat *et al.* takes vertex degree into account, and the measure by Zhang *et al.* is purely weight based. For all results reported in the main manuscript, we used the definition given by Onnela *et al.* (Eqn. 7), but similar results were obtained when using other definitions (see Supplementary Fig. 3 and the associated text for a more extensive discussion on choosing a weighted clustering coefficient).

### Path length

The characteristic path length for a network is given by


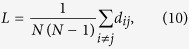


where for a binary network, *d*_*ij*_ is the shortest path between nodes *i* and *j*.

The proper choice of defining *d*_*ij*_ in weighted networks once again depends on the properties of the system. In networks like transportation networks where edge weights represent physical distances, defining *d*_*ij*_ as the sum of the edge weights connecting two vertices is a reasonable choice^62^. However, in many networks edge weights correspond to other types of interactions and it becomes more appropriate to calculate shortest paths based on a specified cost function^63^. In the case of structural brain networks, edge weights represent the density of connections between brain regions. One can think about information flowing between regions along these connections. If there is a high density of connections (large edge weight) between two brain regions, it is intuitive that information could flow more easily between them. We might wish to represent that ease by associating a lower cost to moving between these regions than the cost that we might assign to moving between two regions with a low density of connections between them. For functional brain networks, a high edge weight indicates that the activity time series of two brain regions are close in similarity space. Again, we might wish to represent that region pair’s short distance in similarity space with a low cost of moving between them. This leads to an inverse relationship between edge weight and cost, therefore, as in^64^, we define the distance between two nodes to be *d*_*ij*_ = 1/*w*_*ij*_.

### Bounding of SWP

Because it is possible for real-world networks to display path lengths or clustering coefficients that exceed that of a lattice or random network, we bound Δ_*C*/*L*_ between 0 and 1. Thus, if Δ_*C*_ or Δ_*L*_ > 1, we set Δ_*C*_ or Δ_*L*_ = 1 and if Δ_*C*_ or Δ_*L*_ < 0, we set Δ_*C*_ or Δ_*L*_ = 0, which guarantees that *ϕ* is bounded in the range [0, 1].

### Synthetic benchmark networks

The synthetic benchmark networks were created using the Brain Connectivity Toolbox (BCT)^65^ with modifications to make networks symmetric and with weighting schemes as defined in Lohse *et al.*^28^. All synthetic networks have *N* = 1024 nodes.

Heirarchical modular networks have been used to study features of network topology^66^, and the hierarchical modular small-world networks (HN) used here^67^ were created from the makefractalCIJ function provided in the BCT, with *mx*_*lv* = 10, *E* = 2, and *sz*_*cl* = 5, 6, or 7 for low, medium, and high density networks, respectively. This results in networks with 6, 5, or 4 hierarchical levels with a base module size of *n* = 32, 64 or 128. At the lowest level of the hierarchy, modules are fully connected, and connections are placed within each hierarchical level with a probability *p* = 2^−*l*^, where *l* is the hierarchical level. The weight of each connection *w*_*ij*_ = *p*_*ij*_, such that the weight of a connection is equivalent to the probability that a connection exists. Because this method creates a directed network, the matrices were symmetrized by selecting the upper triangle of the resultant matrix and using these connections to create an undirected, symmetric network. This procedure resulted in final networks with weighted (binary) densities of 4.5(10.8)%, 9.1(18.7)%, and 17.9(31.3)% for low, medium, and high density HN.

Modular small-world networks (MN) were created from the makeevenCIJ function provided in the toolbox, with *N* = 1024, *K* = 65000, 100000, or 150000, and *sz*_*cl* = 6 for low, medium, and high density networks, respectively. This creates networks with 16 fully connected modules of size *n* = 64 and *E* = *K* − 64512 randomly distributed edges between modules. Connection strengths within modules were set to *w*_*ij*_ = 1 while strengths of inter-module edges were set to *w*_*ij*_ = 0.5. As with the HN, this method creates a directed network, so the matrices were symmetrized by selecting the upper triangle of the resultant matrix and using these connections to create an undirected, symmetric network[Fig f6]. This procedure resulted in final networks with weighted (binary) densities of 6.1(6.2)%, 7.9(9.5)%, and 10.3(14.3)% for low, medium, and high density MN.

## Additional Information

**How to cite this article**: Muldoon, S. F. *et al.* Small-World Propensity and Weighted Brain Networks. *Sci. Rep.* **6**, 22057; doi: 10.1038/srep22057 (2016).

## Supplementary Material

Supplementary Information
