# Supplementary material for: Small-World Propensity and Weighted Brain Networks
Source: Sci Rep. 2016 Feb 25;6:22057. doi: 10.1038/srep22057 (PMC4766852; doi:10.1038/srep22057)
Supplement: Supplementary Information [file srep22057-s1.pdf]

Supplementary Information for "Small-World Propensity and Weighted Brain Networks"

Sarah Feldt Muldoon[1,2,3], Eric W. Bridgeford[1,4], and Danielle S. Bassett[1,5,*]

[1]Department of Bioengineering, University of Pennsylvania, Philadelphia, PA 19104 USA
[2]US Army Research Laboratory, Aberdeen Proving Ground, MD 21005, USA
[3]Department of Mathematics and Computational and Data-Enabled Science and Engineering Program, University at Buffalo, Buffalo, NY 14260 USA
[4]Department of Biomedical Engineering, Johns Hopkins University, Baltimore, MD 21218 USA
[5]Department of Electrical and Systems Engineering, University of Pennsylvania, Philadelphia, PA 19104 USA
[*]dsb@seas.upenn.edu

Effects of Density on Clustering and Path Length

As discussed in the main manuscript and shown in Supplementary Fig. 1 for a Watts-Strogatz network, the range of values spanned by the path length and clustering coefficient decreases with increasing network density. Here we define the range of clustering or path length to be the difference between its value in lattice and random networks, $1-C(p=1)/C(p=0)$ or $1-L(p=1)/L(p=0)$. In the main manuscript, we present results showing calculations of the SWP and small-world index for network densities in the range $0-20\%$. At a network density of 20%, we can see that the path length only spans approximately 30% of its original range. Furthermore, by the point at which the density increases to 40%, the path length is the same in lattice and random networks. This implies that the concept of a Watts-Strogatz small-world network is ill-defined for these networks of higher densities, and caution should be used when trying to impose this formalism on high density networks. The effect of network density on path length and clustering further increases the need to take edge weights into account when quantifying network structure. If a network with many weak connections is binarized, the density of the network can become quite high which influences measurements of network properties.

Mapping real-world data to the theoretical model

In order to quantify small-world structure in real-world networks, it is necessary to define a method for mapping the observed data to the theoretical model used to generate small-world networks. Specifically, the calculation of the SWP relies on the generation of a comparable lattice network ($p=0$ in the theoretical models) and a comparable random network ($p=1$ in the theoretical models). To control for network density when generating these comparable null models, we preserve the number of nodes and the distribution of edge weights. As described in the main text, to construct a comparable weighted lattice, we build a 1D network such that the edges that correspond to the smallest Euclidean distance between nodes are assigned the highest weights, whereas to construct a comparable random network, the observed edge weights are randomly distributed among the nodes. In Supplementary Fig. 2, we give an example of the resulting comparable lattice and random networks for the Human DSI data set analyzed in the main manuscript. In the comparable lattice network, the connections with the highest strength have been distributed along the diagonal of the adjacency matrix, while in the comparable random network, connections are randomly assigned throughout the matrix. In both cases, we ensure that the resulting adjacency matrix is free of self-connections and remains symmetric (reflecting the undirected nature of our initial network).

SWP using alternative methods of weighted clustering

Multiple methods of computing a weighted clustering coefficient have been proposed,[1–3] and in the main manuscript we present results using the weighted clustering coefficient as defined by Onnela et al.[1] (see Methods). This particular algorithm for computing the weighted clustering coefficient reflects subgraph intensity and has the advantage of being computationally efficient. However, other definitions of a weighted clustering coefficient exist and reflect other features of weighted clustering that might be desirable in certain data sets.[4] Here, we show the transition in and out of the small-world regime for the same weighted small-world network depicted in Fig. 3 of the main manuscript, along with the corresponding SWP, calculated for the weighted clustering coefficient defined by Barrat et al.[2] (Supplementary Fig. 3a-b) and Zhang et al.[3] (Supplementary Fig. 3c-d). The resultant weighted small-world network displays a similar transition through the small-world regime when measured using

these alternative weighted clustering measures, and the associated SWP is remarkably similar to that of the SWP obtained using the Onnela measure (Fig. 3b-c).

References

1. Onnela, J.-P., Saramäki, J., Kertész, J. & Kaski, K. Intensity and coherence of motifs in weighted complex networks. *Physical Review E* **71**, 065103 (2005).

2. Barrat, A., Barthelemy, M., Pastor-Satorras, R. & Vespignani, A. The architecture of complex weighted networks. *Proceedings of the National Academy of Sciences* **101**, 3747–3752 (2004).

3. Zhang, B. & Horvath, S. A General Framework for Weighted Gene Co-Expression Network Analysis. *Statistical Applications in Genetics and Molecular Biology* **4** (2005).

4. Saramäki, J., Kivelä, M., Onnela, J.-P., Kaski, K. & Kertész, J. Generalizations of the clustering coefficient to weighted complex networks. *Physical Review E* **75**, 027105 (2007).

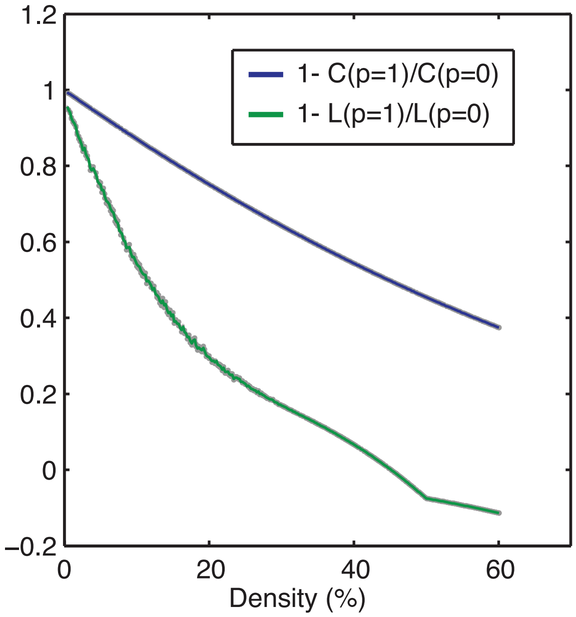

Figure 1. Effects of density on clustering and path length. The range of the clustering coefficient (blue) and path length (red) corresponding to a Watts-Strogatz network for increasing densities (increasing r). Error bars are shown in gray and represent the standard error of the mean calculated over 50 simulations.

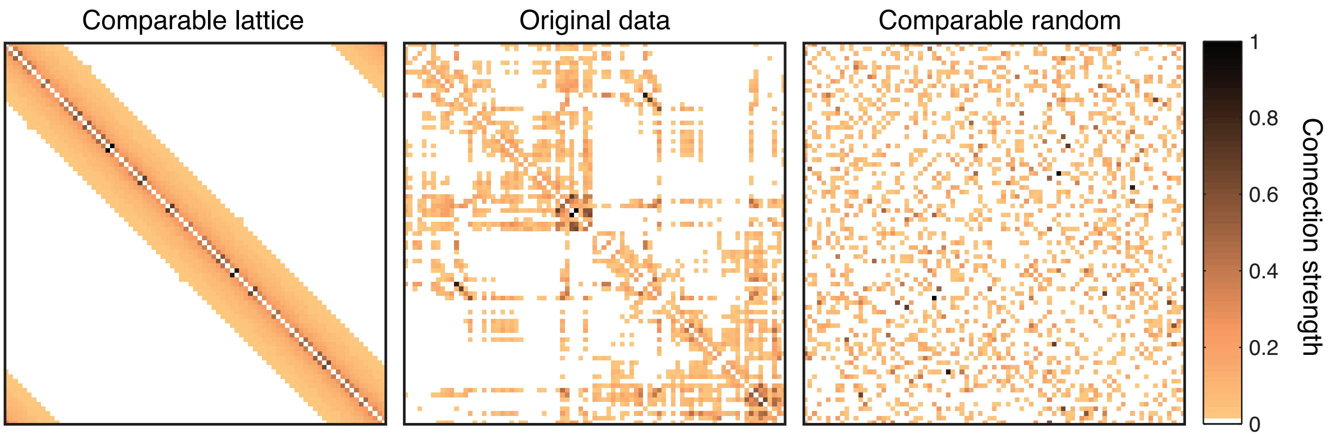

Figure 2. Mapping real-world data to the theoretical model. Adjacency matrices representing the mapping of the Human DSI data from the main manuscript (middle) to a comparable lattice (left) and random (right) network. In both the comparable networks, the original edge distribution is maintained, but connections are redistributed to create a weighted lattice or random network.

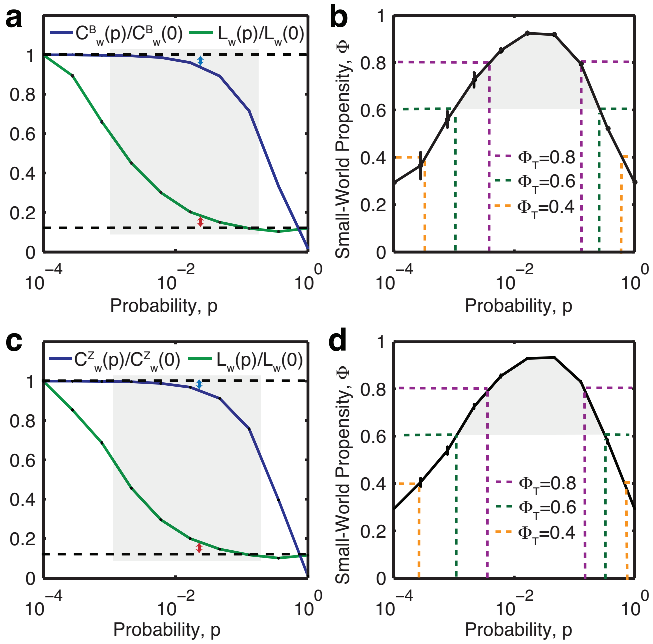

Figure 3. SWP using alternative methods of weighted clustering. (a) Weighted clustering coefficient as defined by Barrat et al. and weighted path length as a function of the rewiring parameter, p, for a weighted formulation of a small-world network with $N = 1000$ nodes and $r = 5$. (b) Weighted SWP calculated using the clustering in (a). (c) Weighted clustering coefficient as defined by Zhang et al. and weighted path length as a function of the rewiring parameter, p, for a weighted formulation of a SWN with $N = 1000$ nodes and $r = 5$. (d) Weighted SWP calculated using the clustering in (c) . Error bars represent the standard error of the mean calculated over 50 simulations, and the shaded regions represent the range denoted as small-world if using a threshold value of $\phi_T = 0.6$. (a) and (c) are comparable to Fig. 3b in the main manuscript and (b) and (d) are comparable to Fig. 3c.
